# Human Exposure to Micro- and Nanoplastics and Their Potential Neurological Implications: A Systematic Review of Emerging Evidence

**DOI:** 10.7759/cureus.110087

**Published:** 2026-06-02

**Authors:** Poonam Parihar, Sindhuprava Rana, Roshan F Sutar, Amit Agrawal, Vijender Singh, Megha K Pandey

**Affiliations:** 1 Translational Medicine, All India Institute of Medical Sciences, Bhopal, Bhopal, IND; 2 Biostatistics, National Institute for Research in Environmental Health, Bhopal, Bhopal, IND; 3 Psychiatry, All India Institute of Medical Sciences, Bhopal, Bhopal, IND; 4 Neurosurgery, All India Institute of Medical Sciences, Bhopal, Bhopal, IND; 5 Neurosurgery, Narayana Medical College Hospital, Nellore, IND

**Keywords:** humans, microplastics, nanoplastics, neurological effects, systematic review

## Abstract

The growing prevalence of micro- and nanoplastics (MNPs) in the environment elicits concerns about their possible impact on human neurological health. Although studies on animals have suggested neurotoxic effects, evidence from humans is still scarce. This systematic review gathers existing human data to assess the presence, types, detection techniques, and neurological consequences of MNPs in different biological matrices.

A comprehensive review was performed on peer-reviewed research concentrating on human studies that report the detection of MNPs in biological tissues and fluids. Four qualifying studies were identified: one clinical observational study, two cadaveric analyses, and one quasi-experimental trial. The data collected encompassed demographics, detection methods, types and concentrations of polymers, biological matrices examined, and neurological biomarkers. MNPs were observed in cerebrospinal fluid (CSF), faeces, urine, olfactory bulbs (OBs), and in brain, liver, and kidney tissues from postmortem cases. The polymers that were reported most frequently were polyethylene (PE) and polypropylene (PP). The detection methods included micro-Fourier transform infrared spectroscopy (µFTIR), pyrolysis-gas chromatography/mass spectrometry (Py-GC/MS), laser direct infrared imaging (LDIR), scanning electron microscopy (SEM), and transmission electron microscopy (TEM).

Although the available evidence is limited, emerging findings indicate the possible accumulation of MNPs in the human central nervous system (CNS), particularly in individuals with dementia or compromised blood-brain barrier (BBB) integrity. Relationships were noted between MNP exposure and disruptions in the BBB, inflammatory markers, and alterations in the gut-brain axis.

This review consolidates the findings and emphasizes the need for further exploration of human exposure to MNPs and their possible accumulation in neural tissues. Although there is variability in methodologies used in the reviewed articles, PE and PP stand out as the primary polymers of concern. While a direct causal relationship cannot yet be confirmed, the results highlight the necessity for improved detection methods, larger sample sizes, and long-term studies to better understand the impact of MNPs on neuroinflammation and neurodegeneration.

## Introduction and background

In 2018, global plastic production reached 300 million tons, and it is likely to continue growing [1,2]. Plastic materials undergo mechanical stress, chemical weathering, photo-oxidation, and biological degradation once they enter the environment, breaking down into microscopic fragments known as microplastics (MPs) [3]. Based on size, plastic waste in the environment is divided into four categories: macro-plastics (>25 mm), meso-plastics (25-5 mm), micro-plastics (5 mm-0.1 µm), and nano-plastics (<0.1 µm) [4].

The pervasiveness of plastics in daily life means that humans are unavoidably exposed to MPs regularly [5]. Ingestion of MPs through food, water, and dust/soil particles; inhalation of indoor and outdoor air; and dermal exposure through cosmetics, textiles, and atmospheric deposition are the three main pathways of human exposure [6].

Recent research has found micro- and nanoplastics (MNPs) in a variety of animals across different environments, such as fish, mice, chickens, and zooplankton [7-9]. MPs have also been detected in human biomonitoring studies, raising concerns about their potential effects on human health. Human absorption and accumulation of MPs, especially in the smaller size range (<50 µm), in critical tissues and organs such as blood, ovaries, testes, placenta, spleen, liver, colon, and lungs, has been documented in some studies [10,11].

Nonetheless, the WHO states that there is currently little evidence that MPs have a major negative impact on human health [12]. There is greater resistance of the central nervous system (CNS) to pathogens and harmful substances. Preclinical research has shown, however, that some MNPs can penetrate the blood-brain barrier (BBB) and accumulate in the brains of mice or fish following injection or ingestion [13,14].

The BBB is a specialized protective interface that regulates the passage of substances from the bloodstream into the brain; disruption of its integrity may facilitate the entry of potentially harmful particles, including MNPs, into the CNS. These findings suggest that MNPs may cross the BBB in animal models, potentially resulting in neurotoxicity and impaired brain function. However, whether MNPs can penetrate the human BBB and enter the CNS is not directly supported by clinical data [15].

Recent advances in analytical techniques, including micro-Fourier transform infrared spectroscopy (µFTIR), pyrolysis-gas chromatography/mass spectrometry (Py-GC/MS), and laser direct infrared imaging (LDIR), have enabled the detection and characterization of MNPs in human tissues and biological fluids. These methods identify polymer composition and quantify plastic particles, thereby facilitating human biomonitoring studies.

Although numerous reviews have summarized the environmental occurrence and experimental toxicity of MNPs, comparatively little attention has been given to evidence derived directly from human studies, particularly regarding the nervous system. Therefore, this systematic review focuses on the current state of research on the neurotoxicity of MPs and highlights their detection in human biological samples.

In particular, we conduct a systematic analysis of clinical studies conducted to date that investigate the association between MP exposure and neurotoxic outcomes in humans. This synthesis aims to qualitatively and quantitatively assess existing evidence on how MNPs may contribute to neurological impairments, including cognitive decline, neuroinflammation, and neurodegenerative diseases in humans. 

Objectives

The objective of the current review was to evaluate the available human evidence on MNP exposure (ingestion, inhalation, and dermal absorption) and explore its potential neurological implications, including reported associations with neurotoxicity, neuroinflammation, cognitive decline, and neurodegenerative diseases in humans.

## Review

Methods

Study Plan

The Preferred Reporting Items for Systematic Reviews and Meta-Analyses (PRISMA) guidelines were followed in conducting the present review. The review focused on evidence of MNP exposure and neurological effects in humans. The protocol was designed for the search strategy, inclusion criteria, and data extraction.

Literature Search Approach

We searched electronic databases, including PubMed, Scopus, the Cochrane Central Register of Controlled Trials (The Cochrane Library), and ScienceDirect, using the search terms outlined in Appendix A. In addition to these databases, the reference lists of included studies were reviewed to identify potentially relevant studies. Two investigators independently screened the abstracts of the shortlisted articles, and the full texts of these articles were evaluated according to the inclusion and exclusion criteria. Conflicts were resolved by consensus, and studies were finalized.

Eligibility Criteria

Eligibility criteria were developed using the PECO (Population, Exposure, Comparator, Outcome) framework. Population (P): human participants of any age or sex; Exposure (E): exposure to or detection of MNPs through ingestion, inhalation, or dermal absorption, or measurement in human biological samples; Comparator (C): unexposed individuals, lower-exposure groups, control groups, or within-subject comparisons when available; Outcome (O): neurological findings including neurotoxicity, neuroinflammation, cognitive impairment, neurodegenerative disease, BBB dysfunction, CNS accumulation of MNPs, or related neurological biomarkers.

Inclusion criteria: Studies conducted in humans that reported exposure to or detection of MNPs and evaluated neurological outcomes, neurological biomarkers, BBB integrity, CNS accumulation, or factors relevant to neurological health were included.

Exclusion criteria: Studies on animals or in vitro experiments, studies describing environmental variables alone, studies combined with MP additives and other substances, studies not related to neurological effects, reviews, chapters, editorials, surveys, conference abstracts, and studies published in non-English literature were excluded. The characteristics of excluded studies are provided in Appendix B.

Data Extraction and Synthesis

Data were extracted using a pre-designed proforma based on the eligibility criteria. The extracted information included study authors, year of publication, country, study design, participant characteristics, sample size, biological matrix analysed, exposure assessment, MP detection techniques, concentrations detected, neurological outcomes, follow-up duration, and other relevant variables. Two reviewers independently verified the extracted data, and discrepancies were resolved through discussion and consensus. Authors were contacted when additional information or clarification was required. Due to the limited number of eligible studies and substantial methodological heterogeneity in study design, biological matrices, detection methods, and outcome measures, a quantitative meta-analysis was not feasible. Therefore, findings were synthesized narratively and presented descriptively according to study characteristics, exposure assessment, and reported neurological findings.

Risk of Bias and Quality Assessment

The methodological quality and risk of bias of the included studies were assessed using the Joanna Briggs Institute (JBI) critical appraisal tools: the JBI Checklist for Case Series [16] for the case series study, the JBI Checklist for Analytical Cross-Sectional Studies [17] for the autopsy and clinical observational studies, and the JBI Checklist for Quasi-Experimental Studies [18] for the dietary intervention study. Two investigators independently evaluated each study to ensure objectivity.

Results

Study Selection

**Figure 1:**
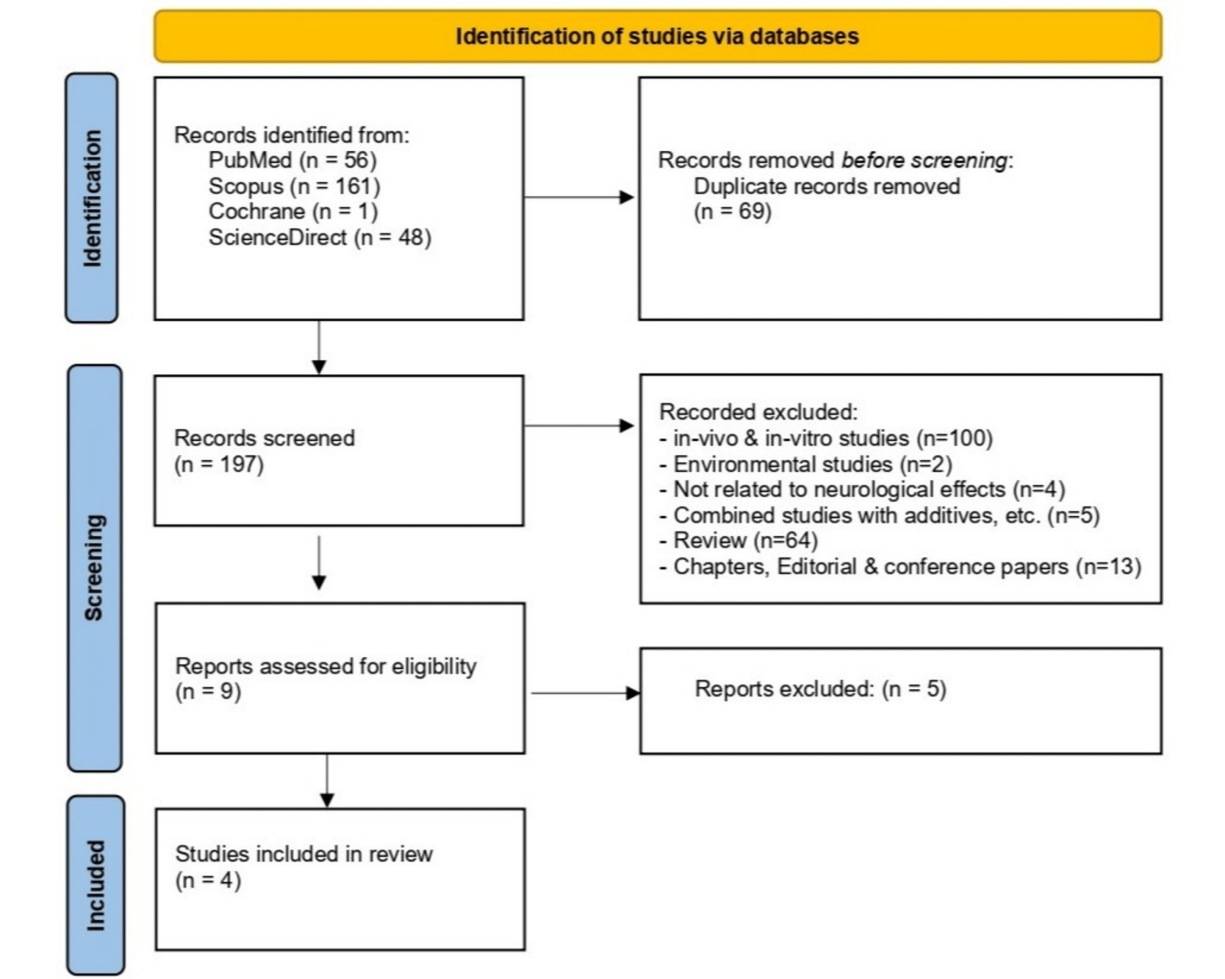
PRISMA chart denoting the articles selection algorithm n represents the number of studies.

This review includes studies on humans that presented the detection and quantification of MPs and the associated neurological outcomes. Searching PubMed, Cochrane, Scopus, and ScienceDirect databases from their inception to April 28, 2025, resulted in 266 manuscripts. After thoroughly applying all inclusion and exclusion criteria and screening for any missing manuscripts, a total of four manuscripts were deemed suitable for inclusion. The article selection algorithm is shown in Figure 1. We provide a description of the characteristics of all studies in Table 1.

**Table 1:** Characteristics of included studies MPs: Microplastics; MNPs: Micro- and nanoplastics; PE: Polyethylene; PP: Polypropylene; PS: Polystyrene; PVC: Polyvinyl chloride; PET: Polyethylene terephthalate; PMMA: Polymethyl methacrylate; PLA: Polylactic acid; DPT: Disposable plastic tableware; OB: Olfactory bulbs; CSF: Cerebrospinal fluid; μFTIR: Micro-Fourier transform infrared spectroscopy; FTIR: Fourier transform infrared spectroscopy; ATR-FTIR: Attenuated total reflectance-Fourier transform infrared spectroscopy; Py-GC/MS: Pyrolysis-gas chromatography/mass spectrometry; LDIR: Laser direct infrared imaging; SEM: Scanning electron microscopy; LC-MS: Liquid chromatography-mass spectrometry; BBB: Blood-brain barrier; CNS: Central nervous system

Study authors (year)	Country	Study type	Sample size	Participants	Type of sample analysed	Exposure group age	Sex	Inclusion criteria	Exclusion criteria	Intervention groups	Technique used for MP detection	Concentration of MPs detected	Outcomes	Follow-up	Remarks
Amato-Lourenço et al. (2024) [19]	Brazil	Case series study (autopsy)	n = 15	Deceased adult individuals undergoing a coroner's autopsy	Bilateral OBs	Median age: 69.5 (range 33-100)	12 Male/3 Female	Residents of Sao Paulo for >5 years; undergoing routine coroner autopsies.	Cases in which the deceased had previously undergone neurosurgical interventions were not selected for the study.	No interventions; observational assessment	μFTIR	1-4 MPs per OB in 8/15 cases	Detection of MNPs in OB, mostly PP and nylon	Single time-point sample collection	First evidence of MPs in OB; possible neurotoxicity
Nihart et al. (2025) [20]	USA	Retrospective cross-sectional autopsy study	20-28 per organ/timepoint; 12 dementia cases; 28 East Coast brains	Deceased individuals and brain banks	Brain (frontal cortex), liver, kidney	52.8 ± 34.3	Both sexes	Postmortem organ availability; normal or dementia diagnosis; years 1997-2024	None specified; limited to de-identified autopsy samples	No interventions; observational assessment	Py-GC/MS, SEM, ATR-FTIR	26,076 µg/g (median) in dementia brains	High MNPs in the brain, especially dementia; PE predominant; evidence of vascular & immune cell accumulation	single time-point sample collection	Trend of increased MNPs in brain; strongest in dementia
Xie et al. (2024) [15]	China	Human clinical observational study	n = 28	Patients undergoing lumbar puncture for neurological evaluation (with or without CNS infection)	CSF	51.75 ± 17.0	Both sexes	Patients undergoing lumbar puncture for diagnostic purposes; diagnosed with or without CNS infections; consented for sample use.	Refusal to participate; low intracranial pressure preventing lumbar puncture.	No interventions; observational assessment	Py-GC/MS and LDIR	PP and PS in all 28 CSF samples, PE in 26 samples, and PVC in 17 samples	MNPs in CSF; PE & PP associated with BBB impairment	Single time-point sample collection	First clinical evidence of MNPs crossing BBB; no inflammation found
Zhang et al. (2024) [21]	China	Quasi-experimental human study	n = 60	Postgraduate students	Faeces, Urine	Exposure: 26.15 ± 0.23; Control: 26.11 ± 0.36	Both sexes	Healthy postgraduate students; willing to consume specified meals packaged in DPT or non-DPT for 1 month.	Diagnosed with diabetes, ulcerative colitis, Crohn's disease, infectious diseases; history of chemotherapy, radiotherapy, or surgery within 3-6 months; abnormal bowel movements a week prior; menstruating women.	Experimental - dietary DPT exposure and withdrawal	LDIR and LC-MS	Exposure: 24.650 items/g; post-exposure 9.805 items/g	Increased MPs altered gut microbiota & metabolites related to CNS	1-month exposure + 1-month post-exposure	DPT-linked MPs affect gut-CNS axis; reversible post-exposure

Study Characteristics

Across the four studies, participants varied widely in age, with the majority being adults or elderly individuals (for example, the median age was 69.5 years in the autopsy series, and there were no significant age differences between the case and control groups in the other three studies). The sex distribution indicated a slight male predominance in some cohorts (for instance, 12 out of 15 deceased individuals were male), although none of the studies found significant differences based on sex between the experimental and control groups. Notably, all studies reported no significant disparities in essential demographic variables such as age, sex, BMI, and habits related to plastic use between the case and control groups, which helps reduce potential confounding factors.

However, studies involving clinical populations, particularly patients with CNS infections, indicated notable increases in inflammatory and cerebrospinal fluid (CSF) markers (e.g., CSF IL-6, IL-8, albumin, IgG, CSF-AI), suggesting underlying pathophysiological changes despite similar demographics. These findings suggest that MP exposure studies may have accounted for basic demographic variables and highlight the importance of incorporating clinical biomarkers to better understand the neurological implications linked to MP exposure.

Results of Individual Studies

The first evidence of MNPs in the human olfactory bulb (OB) was shown by Amato-Lourenço et al., who found particles in 8 out of 15 cases [19]. Sixteen synthetic polymer particles and fibres were found in the investigation, with polypropylene (PP) being the most common (43.8%), followed by nylon and polyamide. In a similar vein, MNPs were found to accumulate significantly in the brain’s frontal cortex by Nihart et al. [20]. Using Py-GC/MS, they discovered that MNP concentrations were much greater in dementia patients’ brains (median 26,076 µg/g) than in healthy brains, with polyethylene (PE) being the most common polymer observed.

Xie et al. detected MNPs in all 28 CSF samples analysed [15]. While PE was detected in 26 samples and polyvinyl chloride (PVC) in 17 samples, PP and polystyrene (PS) were detected in all samples. Significantly, this study found a positive association between the concentrations of PE and PP detected and the CSF-albumin index (CSF-AI), indicating that the entry of these polymers into the CNS is facilitated by reduced BBB integrity.

Zhang et al. showed that MNP load might be reduced by dietary intervention [21]. Faecal MNP concentrations in their quasi-experimental study dropped from 24.650 items/g to 9.805 items/g after participants ceased using single-use plastic tableware. This decrease was accompanied by changes in the gut microbiota, particularly in the abundance of Actinobacteria, Proteobacteria, Firmicutes, and Bacteroidota, as well as metabolites linked to inflammation and the CNS, indicating a possible route by which MNPs could affect neurological health through the gut-brain axis.

Risk of Bias and Quality Assessment

Overall, the methodological quality was high across all included studies, as these studies utilized valid and reliable methods for MP detection (e.g., Py-GC/MS, LDIR, µFTIR) and appropriate statistical analyses (Appendix C) [15,19-21].

Discussion

**Figure 2:**
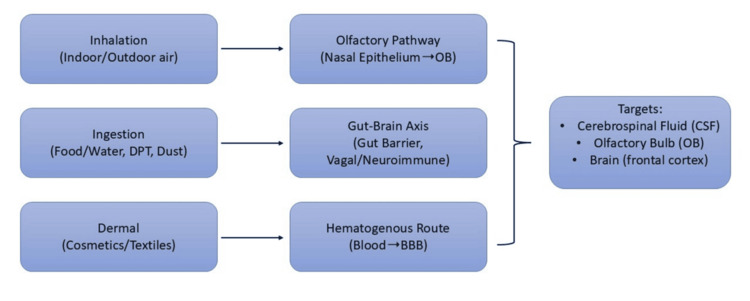
Entry routes and targets for human micro/nanoplastic exposure

Since refractory MNPs can accumulate over time and cause long-term exposure, they pose a serious threat to all living organisms [21]. Although the precise mechanism by which MPs impact human health is unknown, it is possible that they act as conduits for harmful chemicals and viruses from the environment to enter the human body (Figure 2). Numerous studies have been conducted on their effects on the environment and marine organisms [22], but there are not many on humans, and those that have been conducted have reported varying characteristics. Gaps and variations in research and analytical methods could be the cause of this diversity in findings [11]. There has been a dramatic rise in toxicological research on MPs. MNPs have recently been the subject of investigations into their neurotoxic potential, emphasizing their capacity to induce oxidative stress, neuroinflammation, and behavioural changes. However, there is still much to learn about the effects of MNPs in terms of size, type, and dose, especially concerning their involvement in the aetiology of neurodegenerative disorders [23,24].

Baseline Characteristics

Across the four studies, participants varied widely in age, with the majority being adults or elderly individuals (for example, the median age was 69.5 years in the autopsy series, and there were no significant age differences between the case and control groups in the other three studies). The sex distribution indicated a slight male predominance in some cohorts (for instance, 12 out of 15 deceased individuals were male), although none of the studies found significant differences based on sex between the experimental and control groups. Notably, all studies reported no significant disparities in essential demographic variables such as age, sex, BMI, and habits related to plastic use between the case and control groups, which helps reduce potential confounding factors. However, studies involving clinical populations, particularly patients with CNS infections, indicated notable increases in inflammatory and CSF markers (e.g., CSF IL-6, IL-8, albumin, IgG, CSF-AI), suggesting underlying pathophysiological changes despite similar demographics. These findings suggest that MP exposure studies have accounted for basic demographic variables and highlight the importance of incorporating clinical biomarkers to better understand the neurological implications linked to MP exposure.

Biological Matrices for MNPs Assessment

The systematic review encompasses four investigations that examined the presence, distribution, and consequences of MNPs using a wide range of biological specimens. Bilateral OBs from human cadavers were examined by Amato-Lourenço et al. to investigate the nasal pathway as a possible means of MNP entry into the CNS [19]. MNPs in CSF and their correlation with BBB integrity were first clinically demonstrated by Xie et al. [15], who evaluated CSF samples from patients with and without CNS illnesses. In order to track consumption, excretion, and metabolic alterations after exposure to meals served in disposable plastic tableware (DPT), Zhang et al. [21] conducted a quasi-experimental study in which they assessed urine and faecal samples from healthy participants. Meanwhile, Nihart et al. [20] carried out a comprehensive autopsy-based analysis of brain (specifically the frontal cortex), liver, and kidney tissues, uncovering organ-specific burdens of MNPs and a higher concentration in dementia cases. Together, these studies underscore the systemic distribution of MNPs and indicate various routes of exposure and accumulation within the human body.

Qualitative and Quantitative Polymer Profiles

Analysis of the selected studies indicates a consistent pattern of MNP presence across various biological compartments, particularly within the CNS, including CSF, frontal cortex, and OB. PE and PP were found to be the most prevalent types of polymers, exhibiting high detection rates and concentrations in samples from the brain and CSF. An autopsy-based study identified 16 synthetic polymer particles and fibres, including PP (most frequent, at 43.8%), nylon, polyamide, and polyethylene vinyl acetate, in the OB of 8 out of 15 individuals. The majority of these particles were irregular fragments or fibres, suggesting an environmental origin and pointing to the olfactory pathway as a likely entry route for airborne MPs.

Temporal analysis of the Py-GC/MS data highlighted a significant increase in MNP accumulation in brain and liver tissues between 2016 and 2024, with brain samples, particularly from dementia cases (including Alzheimer’s disease and vascular dementia), showing the highest concentrations recorded (median: 26,076 µg/g). In these brain samples, PE constituted up to 75% of the overall polymer load, primarily appearing as nanoplastic flakes, while liver and kidney tissues showed considerably lower MNP levels. These findings suggest a preferential accumulation of MNPs in neural tissues, influenced by environmental exposure trends as well as physiological factors such as tissue permeability and clearance mechanisms.

Further analysis using Py-GC/MS confirmed the presence of PP, PE, PS, and PVC in the CSF of all patients, irrespective of their CNS infection status, with PP and PE showing strong correlations with indices of BBB permeability. LDIR imaging detected numerous particles smaller than 100 µm, although PS was detected in lower quantities, indicating potential differences in how various polymers enter or are retained within the CNS. Interestingly, other common environmental polymers such as polyethylene terephthalate (PET), polymethyl methacrylate (PMMA), and polylactic acid (PLA) were not found in CSF samples, which could suggest selective filtration at the BBB or variations in bio-persistence.

In contrast, a quasi-experimental study involving human subjects examined MP excretion and changes in the gut microbiome after stopping exposure to thermally processed food containers. Although no significant differences in baseline MP levels were observed between exposed and unexposed groups, faecal MP levels dropped considerably after the intervention, accompanied by shifts in gut microbiota related to energy metabolism, inflammation, and neurological disorders. This implies that diet could be a modifiable source of MP exposure and may indirectly affect neuroinflammatory processes through the gut-brain axis.

Overall, these findings offer comprehensive evidence that MNPs, notably PE and PP, are increasingly accumulating in human CNS tissues and may enter through olfactory or hematogenous pathways. Although direct causation with neurodegeneration has not yet been established, the strong correlation with dementia cases and alterations in microbiota underscores the pressing need for longitudinal and mechanistic studies to investigate their potential neurotoxic effects. The integration of analytical methods such as Py-GC/MS and LDIR improves the reliability of polymer detection, paving the way for future biomonitoring and risk assessment within clinical populations.

Degradation and Aging of Plastics

Amato-Lourenço et al. highlighted characteristics of weathering in MP particles and fibres by examining clear spectrum variations between ambient MPs and pristine plastic standards [19]. In aged MPs, the µFTIR spectra showed weak or non-existent peaks, indicating chemical deterioration. This finding, however, is limited to a single dataset, emphasizing the necessity of doing additional biomonitoring research to differentiate between pure and environmentally aged MPs due to their possibly distinct bioreactivity and interactions with human tissues.

Detection and Quantification Techniques

Recent advancements in analytical techniques have enabled the detection and characterization of MNPs across diverse biological matrices and human tissues. The determination of particle size has been successfully carried out using microphotographs captured via µFTIR spectroscopy, with tools such as ImageJ (v1.54g, NIH) utilized for precise morphometric analysis. In CSF, a combination of Py-GC/MS and LDIR imaging has successfully detected and quantified common polymers such as PE, PVC, PS, and PP, representing the first study to accomplish this in CSF samples. Likewise, the use of LDIR laser infrared imaging spectrometry on faecal samples (n = 87) has facilitated a non-invasive evaluation of MP exposure in the human gut. These technologies offer promising insights; however, to confirm findings and better assess health outcomes, analytical methods must be further refined and expanded to include larger, more complex populations. Moreover, although key findings have been consistently reproduced across various tissue banks and analytical laboratories, the relatively high variability observed within samples during Py-GC/MS analyses underscores the need to standardize procedures for clinical applications and to improve the precision of assessing temporal and tissue-specific accumulation of MPs, particularly in organs such as the brain.

Neurological Implications of MNPs

Histological studies of brains affected by dementia have identified refractile inclusions suspected to be MNPs, particularly located around areas of inflammation and along the walls of blood vessels. This suggests a spatial relationship between these plastic particles, immune activation, and vascular damage. The co-localization of MNPs with inflammatory responses raises concerns about their potential role in influencing neuroinflammation and microvascular injury, both of which play a critical role in the progression of cognitive decline. Neurodegenerative diseases such as dementia are characterized by specific pathological features, including shrinkage of brain tissue, disruption of BBB integrity, and compromised clearance systems. These biological alterations may enable the accumulation of MNPs in brain tissue, although there is currently no evidence establishing a direct causal link. In addition, no correlation was found between the age of the decedents and total plastic load; however, a nearly 50% increase in brain plastic concentrations over the past eight years was observed. This trend likely mirrors the exponential rise in environmental MNP pollution rather than age-related accumulation, suggesting that external exposure is a key factor influencing internal plastic levels. The mechanisms behind the uptake and transport of MNPs into the brain are not fully understood. However, studies using *Daphnia magna* models suggest that clathrin-mediated endocytosis and macropinocytosis may facilitate intestinal absorption of nanoplastics, possibly resembling lipid transport processes that can bypass the BBB [25]. This raises the possibility that dietary exposure to MNPs might allow their selective transfer into neural tissues, particularly in individuals with compromised BBB function, which is commonly observed in aging and neurodegenerative disorders.

Clinical Practice Implications

The identification of MNPs, specifically PE and PP, in the human CNS and CSF indicates that medical professionals should consider environmental plastic exposure as a potential neurological health modulator. Healthcare professionals should be especially attentive to the environmental histories of patients with neurodegenerative or neuroinflammatory diseases, because MNP accumulation is much higher in patients with dementia and those with impaired BBB integrity. The review identifies dietary intervention as a crucial modifiable risk factor. Clinical recommendations are based on evidence that the removal of single-use plastic dinnerware considerably lowers faecal MNP burdens and restores gut microbiota. In order to potentially reduce systemic inflammation and safeguard the gut-brain axis, doctors and nutritionists can provide patients with practical recommendations to reduce the use of plastic containers for thermally processed meals. These results highlight the importance of incorporating environmental health literacy into clinical practice to address the growing issue of plastic pollution in aging and vulnerable populations, even though standardized clinical diagnostic tests for MNPs are currently unavailable.

Limitations

This systematic review has several inherent limitations. First and foremost, only four studies met the predefined eligibility criteria, because human investigations directly assessing MNP exposure in relation to neurological outcomes remain extremely limited. This reflects the early stage of research in this field and restricts the strength and generalizability of the available evidence.

Although the review mentions the gut-brain axis, a major drawback is that it does not offer a thorough examination of the intricate, bidirectional signalling of the gut-brain-microbiota axis in the context of MNP exposure. This is mainly because there are currently few clinical studies that mechanistically connect MNP-induced dysbiosis to particular neurotoxic outcomes in humans. Additionally, despite our extensive search approach, this analysis lacks information on several significant neurological conditions, including seizures, epilepsy, and Parkinson’s disease. Since there are currently no human studies that specifically examine MNP accumulation or consequences among these patient cohorts, they were essentially excluded from the body of research, creating a significant knowledge gap.

Additionally, the substantial methodological heterogeneity of the included studies, which employ a range of detection techniques such as Py-GC/MS, LDIR, and µFTIR, makes direct quantitative comparisons of plastic concentrations difficult. The data in the existing literature are also geographically biased, as it is limited to specific populations in Brazil, China, and the USA, potentially ignoring differences in MNP exposure driven by regional environmental policies and lifestyles. Last but not least, since human MNP research is still in its early stages, the lack of established protocols for tissue digestion and “blank” control methods across all studies may raise the possibility of reporting bias or environmental contamination, highlighting the need for a widely accepted standardized framework.

## Conclusions

This systematic review demonstrates that there is reason for concern regarding the ability of MNPs to penetrate the human CNS, especially when the integrity of the BBB is compromised. Among the polymers identified, PE and PP appeared as the most common and biologically significant, particularly in situations involving CNS inflammation or infection. Furthermore, evidence from studies analysing faecal samples suggests that dietary sources are a significant and modifiable pathway for MNP exposure, with noticeable decreases in levels following the elimination of contaminated foods. Collectively, these findings highlight the urgent need for standardized and contamination-free analytical techniques such as Py-GC/MS and LDIR, which could serve as promising strategies for the accurate detection and quantification of MPs in biological specimens. These insights provide a crucial foundation for future translational research aimed at understanding the impact of environmental plastic pollutants on neurological health and disease.

This review sheds light on the emerging presence and potential effects of MNPs in the CNS. However, the current evidence is limited by the small number of high-quality clinical studies and significant variability in methodologies. Considering the potential neurotoxic effects of polymers such as PE, PVC, PP, and PS, along with the rising global prevalence of neurodegenerative diseases, these results highlight the urgent need for further research. To establish causal relationships and assess health risks, future research should focus on employing more advanced analytical techniques, longitudinal study designs, and larger, well-defined cohorts. Given the rapidly increasing environmental prevalence of MNPs, there is an urgent need for coordinated global efforts to investigate their potential role in neurological disorders and overall human health impacts.

## Appendices

Appendix A

**Table 2:** Details of search strategy

Database	Search details
COCHRANE	1 Trial matching microplastics nervous system in Title Abstract Keyword
PubMed	(("microplastics"[MeSH Terms] OR "microplastics"[All Fields] OR "microplastic"[All Fields]) AND ("nervous system"[MeSH Terms] OR ("nervous"[All Fields] AND "system"[All Fields]) OR "nervous system"[All Fields]) AND "humans"[MeSH Terms]) AND (1000/1/1:2025/4/28[pdat])
ScienceDirect	Title, abstract, keywords: microplastics nervous system
SCOPUS	TITLE-ABS-KEY (microplastics AND nervous AND system)

Appendix B

**Table 3:** Characteristics of excluded studies

Study author (year)	Reason for exclusion
Ageel et al. (2024) [6]	Environmental observational study (Indoor air analysis)
Al-Mansoori et al. (2025) [25]	Microplastics pollution in drinking water (i.e., tap water and bottled water)
Asif et al. (2024)	Survey study design
Laganà et al. (2025) [26]	Study on cell lines
Zang et al. (2025) [27]	Study on precocious puberty in girls and female mice

Appendix C

**Table 4:** JBI critical appraisal for case series studies Q1. Were there clear criteria for inclusion in the case series? Q2. Was the condition measured in a standard, reliable way for all participants involved in the case series? Q3. Were valid methods used for the identification of the condition for all participants included in the case series? Q4. Did the case series have consecutive inclusion of participants? Q5. Did the case series have complete inclusion of participants? Q6. Was there clear reporting of the demographics of the participants in the study? Q7. Was there clear reporting of clinical information of the participants? Q8. Were the outcomes or follow-up results of cases clearly reported? Q9. Was there clear reporting of the presenting site(s)/clinic(s) demographic information? Q10. Was the statistical analysis appropriate? Source: [16]

Study	Q1	Q2	Q3	Q4	Q5	Q6	Q7	Q8	Q9	Q10	Score (%)	Risk of bias
Amato-Lourenço et al. (2024) [19]	Yes	Yes	Yes	Yes	Yes	Yes	Yes	Yes	Yes	Yes	100%	Low

**Table 5:** JBI critical appraisal for analytical cross-sectional studies Q1. Were the criteria for inclusion in the sample clearly defined? Q2. Were the study subjects and the setting described in detail? Q3. Was the exposure measured in a valid and reliable way? Q4. Were objective, standard criteria used for measurement of the condition? Q5. Were confounding factors identified? Q6. Were strategies to deal with confounding factors stated? Q7. Were the outcomes measured in a valid and reliable way? Q8. Was an appropriate statistical analysis used? Source: [17]

Study	Q1	Q2	Q3	Q4	Q5	Q6	Q7	Q8	Score (%)	Risk of Bias
Nihart et al. (2025) [20]	Yes	Yes	Yes	Yes	Yes	Yes	Yes	Yes	100%	Low
Xie et al. (2024) [15]	Yes	Yes	Yes	Yes	Yes	Yes	Yes	Yes	100%	Low

**Table 6:** JBI critical appraisal for quasi-experimental studies Q1. Is it clear in the study what is the 'cause' and what is the 'effect' (i.e., there is no confusion about which variable comes first)? Q2. Were the participants included in any comparisons similar? Q3. Were the participants included in any comparisons receiving similar treatment/care, other than the exposure or intervention of interest? Q4. Was there a control group? Q5. Were there multiple measurements of the outcome, both pre and post the intervention/exposure? Q6. Was the follow-up complete, and if not, were the differences between groups in terms of their follow-up adequately described and analyzed? Q7. Were the outcomes of participants included in any comparisons measured in the same way? Q8. Were outcomes measured in a reliable way? Q9. Was an appropriate statistical analysis used? Source: [18]

Study	Q1	Q2	Q3	Q4	Q5	Q6	Q7	Q8	Q9	Score (%)	Risk of bias
Zhang et al. (2024) [21]	Yes	Yes	Yes	Yes	Yes	Yes	Yes	Yes	Yes	100%	Low
